# Tracheoesophageal fistula - a complication of prolonged tracheal intubation

**Published:** 2014

**Authors:** M Paraschiv

**Affiliations:** * General Surgery Clinic, ”Bagdasar-Arseni” Emergency Hospital, Bucharest

**Keywords:** trachea-esophageal fistula, prolonged tracheal intubation, conservative treatment, surgical treatment

## Abstract

Tracheoesophageal fistula most commonly occurs as a complication of prolonged tracheal intubation. The incidence decreased after the use of low pressure and high volume endotracheal cuffs, but the intensive care units continue to provide such cases. The abnormal tracheoesophageal communication causes pulmonary contamination (with severe suppuration) and impossibility to feed the patient. The prognosis is reserved, because most patients are debilitated and ventilator dependent, with severe neurological and cardiovascular diseases. The therapeutic options are elected based on respiratory, neurological and nutritional status. The aim of conservative treatment is to stop the contamination (drainage gastrostomy, feeding jejunostomy) and to treat the pulmonary infection and biological deficits. Endoscopic therapies can be tried in cases with surgical contraindication. Operation is addressed to selected cases and consists in the dissolution of the fistula, esophageal suture with or without segmental tracheal resection associated. Esophageal diversion is rarely required. The correct indication and timing of surgery, proper surgical technique and postoperative care are prerequisites for adequate results.

## Introduction

Esophagotracheal and esophagobronchial fistulas represent a pathological entity characterized by the presence of an abnormal communication between the tracheobronchial tree and the digestive tract - the esophagus. The consequences of permanent pulmonary contamination by food containing and digestive secretions can be very serious, with a possible fatal evolution.

The first publications of a post-intubation esophagotracheal fistula are attributed to d' Avignon (1956) and Mounier - Kuhn (1958) [**1**,**2**], and the first prospective study on the incidence and pathogenesis of post-tracheostomy and mechanical ventilation tracheal injury belongs to Andrews and Pearson [**3**]. In this prospective study, over a period of two years, that included 220 tracheostomized, critical patients (of whom 103 survived), two esophagotracheal fistulas developed.

Currently, prolonged intubation is the main cause of benign tracheoesophageal fistula, although the introduction of high volume and low pressure endotracheal tube cuffs reduced the incidence of this complication. The incidence is between 0.3 and 3% in patients with prolonged mechanical ventilation [**4**]. Tracheostomy does not seem to decrease the risk of developing post-intubation esophagotracheal fistula.

**Pathogenesis**

**Fig. 1:**
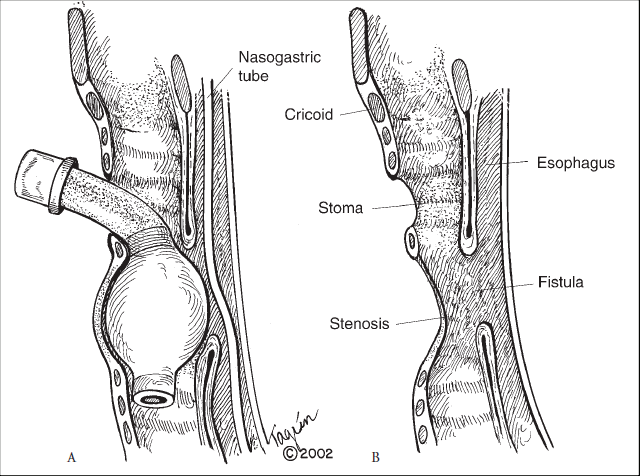
Tracheoesofageal fistula formation in a cannulated patient. The fistula lies few centimeters below the stoma and associates tracheal stenosis. From Grillo HC. Surgery of the tracheal and bronchi, 2004, BC Decker Inc. Hamilton London

Pathogenic mechanism is represented by the chronic trauma of prolonged tracheal intubation. The pressure resulted from the hyper-inflated endotracheal tube cuff on the posterior membranous wall, most often against a rigid nasogastric tube, produces ischemic necrosis that also affects the anterior wall of the esophagus, the result being an abnormal communication. Usually, in these situations, tracheal stenosis co-exist (**Fig. 1**).

Very rarely, fistulas may result from bedsores lying at the tip of a rear-angulated cannula, along with the pressure from a transesophageal rigid probe. In this case, circumferential tracheal injury is missing. If the esophagotracheal fistula is located at the same level with the stoma, the pathogenic mechanism might be a direct tracheoesophageal injury during tracheostomy.

A number of risk factors such as the following are mentioned: high airway pressure during mechanical ventilation, excessive mobility of the endotracheal tube, prolonged time of intubation, steroids treatment, insulin-dependent diabetes, poor nutritional status, chronic hypoxia in cardiopulmonary diseases, prolonged episodes of hypotension, chronic anemia, sepsis and gastro-esophageal reflux. Payne [**5**] reported associated risk factors: female gender and old age.

**Pathology**

The localization of post-intubation fistula is high, in the cranial half of the trachea and, in the case of patients with tracheostomy, 1-2 cm distal from the stoma, at the site of the endotracheal cuff. Left axis deviation of the cervical and upper thoracic esophagus determines the location of the fistula at the left edge of the membranous wall.

The dimensions are variable, but most of the fistulas are “giant” (4-5 cm), over the entire width of the posterior wall being destroyed.

The process of fistula formation is long. Perilesional inflammation binds the tracheal and esophageal walls, so mediastinitis never occurs. Spontaneous healing of the fistula is illusory because the edges are epithelialized. In many cases, fistula formation is associated with circumferential tracheal destruction produced by the same mechanism of ischemic necrosis. This association requires tracheal resection and termino terminal anastomosis [**6**,**7**].

**Clinical presentation**

The clinical manifestations differ, depending on the patient's respiratory status.

**In ventilatory assisted patients:**

- air leaks even with hiperinflated cuff

- abdominal distension associated with ventilation rhythmated air-liquid sounds 

- tracheobronchial contamination with food and digestive secretions (gastric juice, bile)

- broncho-pulmonary suppuration

**In normal breathing and oral feeding patients:**

- ONO`s sign (deglutition followed by cough – in the absence of other swallowing disorders)

- expectoration of food remains and bile coloured secretions

- bronchopulmonary suppuration with respiratory deterioration

Due to their localization (2 cm distal to the stoma), post-tracheostomy fistulas can be observed transstomally after decannulation.

In a cannulated and orally fed patient, the ingestion of methylene blue dye-solution could be a diagnostic (the dye is coughed through the cannula). 

**Imagistic studies**

Chest X-rays can show a dilatation of the esophagus (distal to the fistula) and of the stomach [**8**]. It may highlight the hyper-transparency caused by overinflated endotracheal cuff with a diameter greater than 35 mm (indirect sign of fistula) [**9**]. It can also reveal the radiological signs of secondary pulmonary abscesses.

The esophagram is especially useful where endoscopic examination is not possible. Ingestion of a small amount of contrast agent usually reveals the site of the fistula and the abnormal route of the contrast substance into the tracheobronchial tree. A contrast substance that produces a minimal bronchial inflammation should be used.

**Fig. 2:**
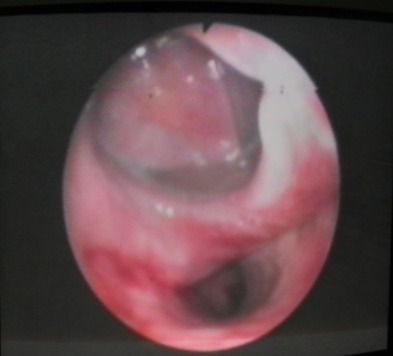
Endoscopic appearance of a huge fistula (bigger than the tracheal lumen). Nasogastric tube can be observed in the esophageal lumen

If a tracheoesophageal fistula is suspected, the most accurate investigation and one that should always be done is bronchoscopy. It identifies the site (in relation with the glottis, cricoid cartilage, carina and with a possible tracheal stoma), the size of the fistula and the length of the normal airway segments. Bronchoscopy also reveals the possible association of circumferential tracheal destruction. The esophageal lumen and a nasogastric tube can be observed through the parietal defect. In case of doubt of the diagnosis methylene blue solution may be instilled on the nasal-esophageal probe (**Fig. 2**).

Esophagoscopy has fewer chances to highlight the fistula (especially when its dimensions are reduced), which may be hidden by the longitudinal mucosal folds.

EBUS-combined echo-endoscopic tracheobronchial investigation provides information about the status of peritracheal tissues.

**Fig. 3:**
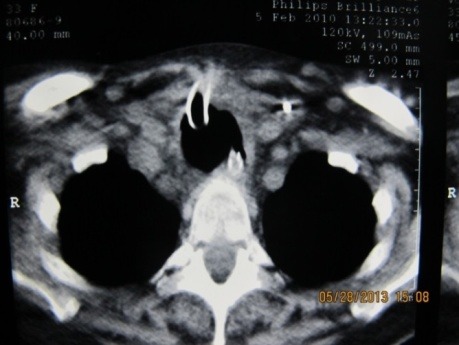
CT scan of a giant trachea-esophageal fistula. Large communication and total destruction of the cartilaginous structure of the trachea

CT scan examination is not required for the diagnosis of tracheoesophageal fistula, but it can confirm the presence of it, the possible associated stenosis and the bronchopulmonary suppuration (**Fig. 3**).

**Therapeutic management**

Treatment is difficult because of the many deficits of patients: poor respiratory status (pulmonary infection, mechanical ventilation), severe biological condition, poor nutritional status and the presence of associated diseases (cardiovascular, neurological).

Optimal time for surgery is established when the patient no longer requires mechanical ventilation and his biological status is improved [**10**-**12**]. Mechanical ventilated patients are not candidates for surgery because of the negative effect of tracheal intubation on anastomosis and esophageal suture, with a very high risk of complications. In these cases, conservative treatment is chosen. The general prognosis will be taken into account. A bad short-term prognosis excludes surgery [**13**-**15**].

**Conservative treatment** is sometimes used as a preparation prior to surgery. The goals are the cease of contamination of the tracheobronchial tree with digestive contain, the resolution of bronchial-pulmonary suppuration, the improvement of the patient`s nutritional status and the weaning from ventilator.

If a nasogastric tube is present, it will be extracted and, in conscious patients, oral feeding will be stopped. A drainage gastrostomy and a feeding jejunostomy will be installed. This can be done by laparotomy, or by minimally invasive surgery (laparoscopic jejunostomy or percutaneous endoscopic combined technique). The patient will be held with the body elevated to 45 degrees at least a few hours after meals. Anti-secretory and prokinetic medication will be associated to prevent gastro-esophageal reflux. The cuff of the tracheostomy cannula will be placed distally to the fistula under endoscopic control and will be inflated with the minimum amount of air that seals the trachea. Repeated bronchial aspiration will be performed. Esophageal diversion is almost never necessary. The small amount of saliva, which may contaminate the tracheobronchial tree, is not usually a problem. Atropine type medication in purposes of minimizing saliva secretion can be administered [**8**,**16**]. In exceptional circumstances, when esophageal diversion is necessary, cervical-esophageal fistula will be terminal and not in continuity (dividing the esophagus immediately proximal to the fistula and the distal end of the esophagus will be closed). Continuous aspiration by gastrostomy is enough to prevent gastroesophageal reflux.

The energetic necessities of an immobilized patient are of about 20-25 kcal / kg / day and 30-35 kcal / kg / day in patients with physical activity. Parenteral nutrition will be temporary used only in addition to the enteral intake, which is mandatory. The healing of the pulmonary suppuration is achieved by antibiotic therapy and respiratory physiotherapy.

Literature mentions sporadic successes by using endoscopic treatment. Clerf et al. showed that it is possible to close the fistula by endoscopic cauterization with small crystals of sodium hydroxide [**17**].

Other endoscopic solutions have been proposed, such as minimal excision of the edges and closure of the esophageal orifice with fibrin glue or clips [**18**,**19**].

Tracheal and/or esophageal stent implantation have been used for the endoscopic treatment of the fistula in patients with surgical contraindication [**20**,**21**].

Tracheal stenting can be taken into account in the following situations: proximal lesion (an esophageal stent in this location would interfere with the upper esophageal sphincter competency), the association of tracheal stenosis due to the destruction of the cartilage, after the previous placement of an endoesophageal stent without sealing of the fistula or if the esophageal stenting is impossible. Also, if there is any concern about an esophageal stent that may reduce (after expanding) the tracheal lumen, the stent will be placed in the trachea. The stent`s length should exceed 2-3 cm cranially and caudally the limits of the fistula.

Rigid silicone stents, very effective in palliation of neoplastic stenosis, have several disadvantages for benign tracheal stenosis: high rate of migration, small inner diameter due to the thickness of the silicone wall, the absence of re-epithelialization and the lack of collapse during cough (which leads to difficulties in the clearance of the secretions). In the 1990s, self-expanding metal stents have replaced rigid plastic prostheses in an attempt to resolve these deficiencies. However, metal stents also have some drawbacks: they can cause tracheal wall perforation with possible vascular lesions, can form exuberant granulomatous tissue with stenosis.

Partially covered metal stents are a newer acquisition. They develop a less granular reaction and can also be easier extracted.

An attractive option for the future could be the biodegradable stents, which have lower migration rates and do not need to be extracted [**22**]. Recently (available in the U.S. since 2003), self-expanding plastic stents were introduced.

In the case of stenting a tracheoesophageal fistula, a covered stent that can be extracted after 3-4 weeks should be used. After the stent extraction, the residual presence of the fistula is checked. If it is not completely healed, another covered stent will be placed for another 4-6 weeks. In 35% of the cases, the tracheoesophageal fistula recurs.

**Surgery**

The objective is to close the fistula with a separate suture of the fistulous orifices. Any area of lung parenchyma with irreversible damage must be resected in the same operative session. In case of simultaneous tracheal stenosis, segmental tracheal resection and termino terminal anastomosis will be associated. In both situations, most authors recommend the interposition of viable tissue between the two sutures, although there are opinions that this is not mandatory. The operation will be performed in a single session as described by Grillo et al. in 1976 [**6**]. This approach is used by other authors [**1**,**2**,**10**,**11**,**23**]. In some cases, the tracheal stenosis may be too long and cannot be resected. After fistula repair, the trachea will be stented with a permanent T- tube. There are sporadic communications of more extensive surgery in such cases. Gallan et al. [**3**] reported a case of eso tracheal fistula associated with a 6.5 cm length tracheal stenosis, in which the authors conducted a membranous wall tracheoplasty by using an esophageal wall after they excluded the esophagus and performed an esophageal - gastric by-pass with colic graft.

**Anesthesia**

Large fistulas may cause problems during anesthesia. Endotracheal tube cuff placement is necessary, preferably under endoscopic control [**24**], between the fistula and the carina, to avoid intraoperative anesthetic gas losses. Anesthesia can be initiated on the usually pre-existing tracheostomy cannula, or the patient can be orotracheally intubated. If tracheal resection is associated, after dividing the trachea distally to stenosis, temporarily intubation through the operative field will be performed.

Continuous suction on the gastrostomy tube will be performed in order to prevent the accumulation of anesthetic gas escaped through the fistula in the stomach. An intra-esophageal tube will placed in order to facilitate intraoperative esophagus marking [**24**].

**Cervical or cervico-mediastinal approach**

Most fistulas are located in the cervical region, at the site of tracheostomy tube cuff, so a transverse or "Y” cervicotomy is usually sufficient for access. The incision can circumscribe the stoma. Very rarely it is necessary to associate partial median sternotomy, if the fistula lies in the thorax.

**Fig. 4:**
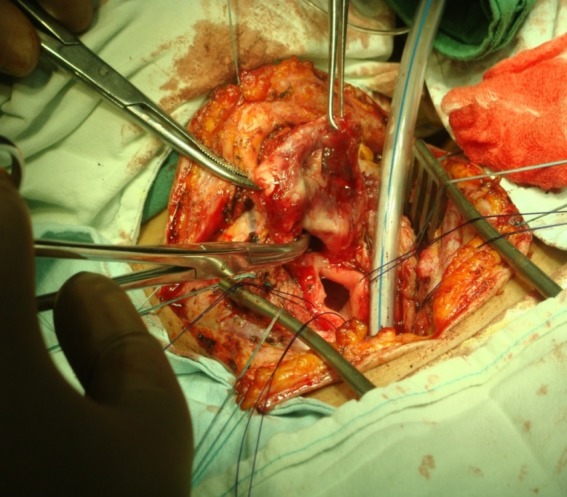
Esophagotracheal fistula associated with complete segmental destruction of trachea both at the site of stoma and at the cannula cuff. Approach by ”Y” cervicotomy. After distal tracheal section, intubation through the operative field is done. Note the tracheal segment to be resected, pulled up from the esophagus. Esophageal orifice opened with traction sutures. Proximal tracheal lumen shown with Kocher forceps

The cranial flap is prepared up to the hyoid bone and the distal one to the sternoclavicular joints. The thyroid isthmus is sectioned between the forceps and a dissection of the thyroid lobes from the trachea is performed. The dissection then progresses in a strictly tracheal plane, thus, recurrent laryngeal nerves (located in the trachea-esophageal grooves) will be avoided. Their dissection and evidence are not necessary or advisable. Tracheoesophageal space is penetrated immediately distal to the fistula. Circumferential dissection of the distal tracheal will not be extended for more than 1-2 cm below the distal plane of the fistula and tracheal stenosis (usually not more than 2 free cartilages), in order not to de-vascularize the remaining trachea. A circumferential dissection of the esophagus is not required but the anterolateral walls must be sufficiently released at the site of the fistula to allow a longitudinal suture of the defect without tension. The high location of the fistula, with the proximal limit near the cricoid, often precludes the posterior dissection at this level, because of the danger of damaging the recurrent laryngeal nerves at the entrance in the larynx. Lateral traction sutures are placed at proximal and distal margins of the tracheal resection. The trachea is sectioned immediately distal to the stenotic segment with intubation through the surgical field. The proximal end of the trachea is pulled and complete lateral dissection of the fistula is made. The fistula is sectioned by preserving as much normal esophageal wall as possible. The proximal end of the fistula is reached by freeing the space between the trachea and esophagus. The trachea is sectioned proximally, above the stenotic segment and the stenotic and fistulized tracheal segment is removed from the surgical field (**Fig. 4**).

**Fig. 5:**
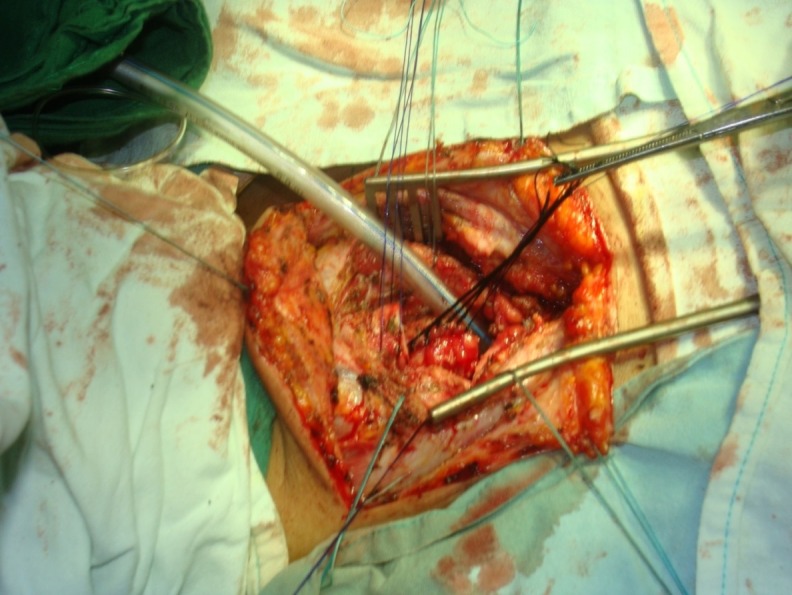
Tracheal resection and esophageal suture completed. Note the normal caliber and structure of the proximal tracheal end

The esophagus is longitudinally sutured in two layers with 4-0 silk or Vicryl (**Fig. 5**).

The first layer brings together the mucosa and submucosa by inverting interrupted sutures in Sweet technique with knots tied on the inner surface. The esophageal muscle sheet is then closed in Lembert fashion. Esophageal suture tightness is checked by the instillation of methylene blue solution through the nasoesophageal tube, withdrawn at that level. Esophageal suture is covered with a pedicled muscle flap made from the sternohyoid muscle, detached from its insertion on the hyoid. The flap is fixed circumferentially around the esophageal suture. Tracheal ends are anastomosed in the usual technique (separate Vicryl 3-0 sutures, with the knots on the outside, starting from the posterior wall). Tension in the anastomosis is reduced by mobilizing the trachea (obtained by blunt dissection of the anterolateral wall) and by cervical flexion. Sometimes laryngeal release procedures could be necessary.

There are the so-called “giant fistulae” in which the fistula is longer than the tracheal segment with circumferential cartilage destruction. In this case, extended tracheal resection, up to the distal point of the fistula, is not recommended. In such cases, only the complete destructed tracheal segment will be resected. Distally to the resected area, the tracheal membranous wall, affected by fistula can be reconstructed by preparing and “borrowing” some of the esophageal wall.

Depending upon the position of the stoma with regard to the stenotic segment, very near or separated by a normal tracheal segment (two or more cartilages), the stoma will be included in the tracheal segment to be resected, or it will be left in place, for not over-extending the limit of the tracheal resection. However, if an inflammatory, granulomatous process affects the intermediate segment, it is more convenient to include it in the tracheal resection.

**Tracheal fistula closure without resection**

If the fistula does not associate tracheal stenosis, the tracheal resection is not required. The cervical approach can be done by transverse or ”Y” cervicotomy, but also by an oblique incision at the anterior border of the sternocleidomastoid muscle. The left side is preferred, because the encircling of the esophagus is easier, the right recurrent laryngeal nerve being more distant from the esophagus than the left one, due to its higher origin. Oblique incision will not be used in case of recurrent fistula. Transverse incision, possibly extended obliquely upward, will be used whenever circumferential tracheal dissection is necessary and especially in re-interventions after failed attempts.

**Fig. 6:**
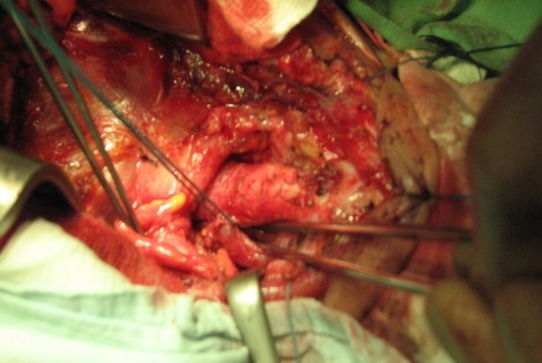
Fistula without tracheal stenosis. Approach by ”Y” cervicotomy and partial sternotomy. Fistula is encircled

**Fig. 7:**
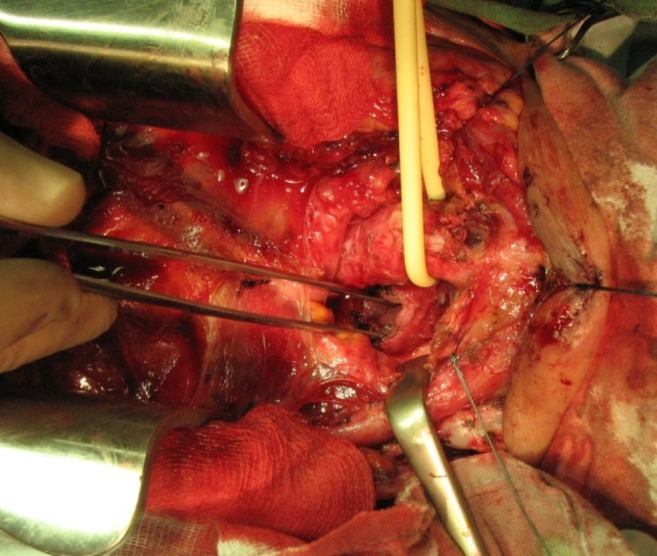
Fistula excision with esophageal orifice opened

**Fig. 8:**
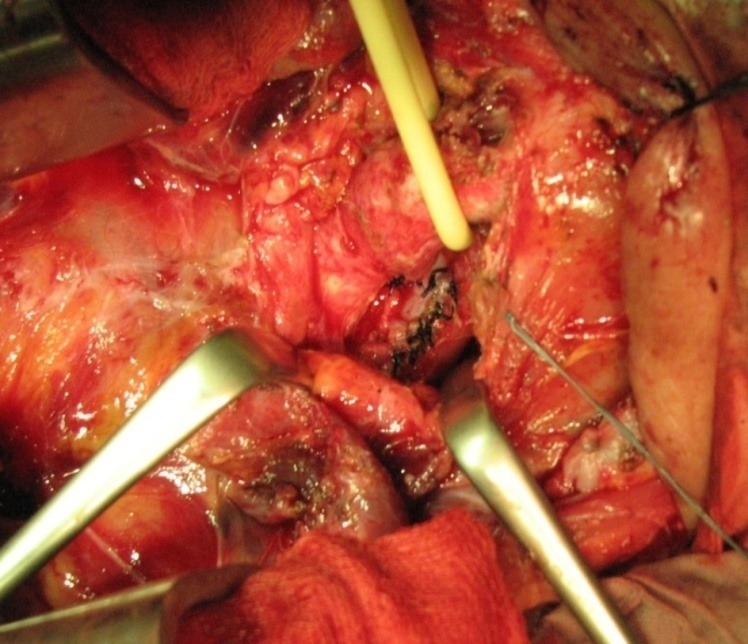
Tracheal and esophageal sutures

After fistula dissection, it will be sectioned, preserving enough membranous tracheal wall, as close to the esophagus as it can be done (**Fig. 6**,**7**). The esophagus is longitudinally sutured by using the described technique and then the posterior membranous tracheal wall (**Fig. 8**).

A pedicle muscle flap is interposed between the two sutures. If the dimensions of the fistula are large, an anterior approach is mandatory, followed by primary tracheal dissection. Sometimes, segmental resection may be tactically necessary in the absence of circumferential cartilage damage, to allow fistula approach.

**Trans-thoracic approach**

In the rare situations of intrathoracic tracheoesophageal fistula, situated immediately above the carina, or broncho-esophageal fistulas, the approach will be a right posterolateral thoracotomy through the fourth intercostal space, after an unilateral left lung intubation. In the left bronchoesophageal fistula, the approach is a left thoracotomy. Azygos vein section provides access to the distal trachea and carina. The esophagus is dissected circumferentially, cranially and caudally from the fistula, and is encircled for traction from both poles. Fistula and the partners are treated according to the technique described and muscle plasty, (using intercostal muscle pedicle flap harvested at the time of thoracotomy), is performed.

**Postoperative Care**

When tracheal resection is associated, the patient will maintain cervical flexion for 7-10 days. The “guardian" suture, between the chin and the presternal skin prevents accidental over-extension. The patients are extubated in the operating room, being then transferred to the intensive care ward. The need for temporary postoperative mechanical ventilation is an important risk factor for anastomotic dehiscence. If ventilatory support is necessary, an endotracheal tube without cuff inflation or a tracheostomy distal to anastomosis will be used. Oral nutrition is prohibited 10-14 days, enteral feeding by jejunostomy tube being performed early (12-24 hours postoperative). The healing is checked two weeks after surgery by contrast study.

## Results

Fistula recurrence after surgery is between 3 to 8.3 % [**10**,**11**,**25**]. Macchiarini et al. [**10**] have observed recurrent laryngeal nerve paralysis in 6.3% of their cases and the recurrence of tracheal stenosis in 14.2%.

Dartevelle and Macchiarini found fistula recurrence between 6.4 and 8.3% and mortality rates between 6.3 and 12.5% [**10**,**25**].

Last series published by Grillo (2004) includes 38 patients of which 27 with post-intubation fistulas. Mortality rate was of 1 in 27. Postoperative vocal cord dysfunction occurred in one case, fistula recurrence after surgery was between 3 to 8.3% [**10**,**11**,**25**].

## Conclusions

Esophagotracheal fistula after prolonged intubation is a severe complication. It occurs in patients with poor nutritional and biological status, with serious associated diseases and dependence for prolonged mechanical ventilation. Pulmonary contamination causes severe broncho-pulmonary suppuration. The treatment is different, whether the patient is mechanically ventilated or not. Conservative treatment cannot cure the injury, but may limit the contamination of tracheobronchial tree also allowing the patient to feed. In most of the cases, surgery associates segmental tracheal resection. The appropriate selection of patients may provide better postoperative outcome premises.
